# Activation of Deoxyribonuclease I by Nicotinamide as a New Strategy to Attenuate Tetracycline-Resistant Biofilms of *Cutibacterium acnes*

**DOI:** 10.3390/pharmaceutics13060819

**Published:** 2021-05-31

**Authors:** Yi-Hsien Shih, Donald Liu, Yen-Chou Chen, Ming-Hsuan Liao, Woan-Ruoh Lee, Shing-Chuan Shen

**Affiliations:** 1 Department of Dermatology, Taipei Medical University Shuang Ho Hospital, New Taipei City 23561 Taiwan; yh.shih@tmu.edu.tw (Y.-H.S.); liudonsen@gmail.com (D.L.); wrlee@tmu.edu.tw (W.-R.L.); 2 Department of Dermatology, School of Medicine, College of Medicine, Taipei Medical University, Taipei 11031, Taiwan; 3 Graduate Institute of Medical Sciences, College of Medicine, Taipei Medical University, Taipei 11031, Taiwan; yc3270@tmu.edu.tw (Y.-C.C.); jcat30132@gmail.com (M.-H.L.); 4 International Master/PhD Program in Medicine, College of Medicine, Taipei Medical University, Taipei 11031, Taiwan

**Keywords:** *Cutibacterium acnes*, biofilm, nicotinamide, deoxyribonuclease, acne vulgaris

## Abstract

Biofilms of *Cutibacterium* (*C.*) *acnes* (formerly *Propionibacterium acnes*) are responsible for the persistence and antibiotic resistance of acne vulgaris. In addition to the standard treatments for acne vulgaris, a common adjunctive treatment is the topical administration of nicotinamide (NAM). However, the effects of NAM on biofilms of *C. acnes* have never been explored. This study comprehensively investigates the effects of NAM against biofilms of *C. acnes* using in vitro and in vivo approaches. The results showed that NAM potentiated the efficacy of suboptimal dosing of tetracycline against *C. acnes*. Moreover, NAM alone decreased the formation and increased the degradation of biofilms in *C. acnes*. The antibiofilm effect of NAM against *C. acnes* was further enhanced in combination with deoxyribonuclease (DNase) I, an enzyme with known antibiofilm properties. The computational molecular docking, surface plasmon resonance analysis, and enzymatic kinetic assay demonstrated that NAM binds to DNase I and accelerated its reaction. In conclusion, NAM activates DNase I to attenuate biofilms of *C. acnes*. This offers valuable insights into the strategies against biofilms that are worth elaborating on in other biofilm-related chronic cutaneous infections in the future.

## 1. Introduction

Acne vulgaris is a common skin disorder that often leads to excess psychological stress and compromises social activities. It causes an enormous loss of 7.2 disability-adjusted life years (DALYs) per million globally [1]. Pathogenesis of the disease is highly associated with colonization of *Cutibacterium* (*C*.) *acnes* (formerly *Propionibacterium acnes*), a Gram-positive aerotolerant anaerobe [2]. The bacterium mostly colonizes sebaceous areas, such as the scalp, face, and upper back [3], producing lipases that metabolize sebaceous triglycerides into propionic acid and free fatty acids that irritate follicular walls and secreting exoenzymes and neutrophil chemoattractants that activate inflammasomes and trigger tissue inflammation [4,5]. Besides lipases, *C. acnes* also expresses other putative virulence factors, including adhesin dermatan-sulfate (DsA1 and DsA2), Christie–Atkins–Munch-Peterson (CAMP) factors, sialidases, endoglycoceramidases, thermal shock proteins, and porphyrin, leading to activation of the innate immune recognition system via TLR2 and TLR4, the NF-κB and MAPK signaling pathways, and the NLRP3 inflammasome pathway [6].

Targeting the bacterium that causes acne vulgaris is critical in treating the disorder, and various antibiotics have been widely used. However, *C. acnes* strains resistant to one or more antibiotics have drastically increased [7]. The overall incidence of *C. acnes* resistance increased from 20% in 1978 to 62% in 1996, with many countries reporting that over 50% of *C. acnes* strains were resistant to topical macrolides [8]. Resistance in *C. acnes* has been associated with reduced response, no response, or a relapse to antibiotics [8], so there is an unmet need for novel acne treatments.

While one way that antibiotic resistance occurs is through genetic mutations, *C. acnes* may also acquire antibiotic resistance by forming a distinct bacterial community called a biofilm. It is well known that biofilms exist in about 65% of chronic infections caused by different pathogens, such as infections by *Pseudomonas aeruginosa* [9], *Staphylococcus aureus* [10], and *Mycobacterium* spp. [11,12]. For these and other pathogens, biofilms were reported to be associated with antibiotic resistance [13]. Likewise, more biofilms of *C. acnes* are found in follicles of acne patients than in those of controls [14], providing a biological glue that holds corneocytes together to form plugs [15] and resulting in increased virulence factors [4] and antiphagocytosis functions [16].

Studies on various pathogens have shown that the formation of biofilms is markedly reduced by deoxyribonuclease (DNase) I [17]. The formation of biofilms usually begins with adhesion, aggregation, microcolony formation, maturation, and dispersion. Most biofilms are composed of mixtures of microorganisms and extracellular polymeric substances, such as polysaccharides, proteins, water, extracellular deoxyribonucleic acid (DNA), and excreted cellular products [17]. Admixtures of the above-mentioned elements are crucial for forming a habitat with a stable architecture, adequate localized gradients of nutrients and waste, and tolerance to and resistance against environment challenges such as antibiotics [13]. DNase I degrades the indispensable component of biofilms, i.e., extracellular DNA [13], and is now in clinical use for treating cystic fibrosis [18].

A potential treatment against biofilms of *C. acnes* is nicotinamide (NAM). Topical NAM is an effective adjunctive therapy for acne vulgaris [19] and was proven to be comparable or even superior to clindamycin in several clinical studies [20,21]. Although NAM is known as a potent inhibitor of proinflammatory cytokines and leukocyte chemotaxis [22], little is known about its detailed mechanism of action in acne vulgaris. The objective of this study was to investigate the effect of NAM on the formation and degradation of biofilms of *C. acnes*. As DNase I possesses known antibiofilm properties, this study also evaluated whether NAM interferes with biofilms of *C. acnes* through DNase I.

## 2. Materials and Methods

Bacterium and Chemicals: The *C. acnes* strain LMG 16711 (ATCC 6919) (Bioresource Collection and Research Center, Hsinchu, Taiwan) was used in all experiments. The bacterium was routinely grown in brain heart infusion (BHI) (BD Bacto™, Sparks, MD, USA) and incubated in anaerobic jars with AnaeroPack-Anaero (Mitsubishi Gas Chemical, Tokyo, Japan) at 37 °C. Chemicals used in this study were as follows: NAM (Sigma-Aldrich, St. Louis, MO, USA), tetracycline HCL (TCN) (BioShop, Burlington, ON, Canada), dextrose glucose (BD Difco™, Sparks, MD, USA), fluorescein isothiocyanate (FITC) (Sigma-Aldrich), Calbiochem FluorSave Reagent (EMD Millipore, Darmstadt, Germany), propidium iodide (PI; Sigma-Aldrich), DNA sodium salt (Sigma-Aldrich), DNase I and reaction buffer (Promega, Madison, WI, USA).

Biofilm Culture System and Biofilm Tests: The biofilm culture system and biofilm tests were modified from an earlier report [23]. The absorbance of the bacterial suspension at 600 nm (A600) was detected by a spectrophotometer to approximate the colony-forming units (CFU)/mL of *C. acnes*. The correlation of absorbance to CFU/mL was good for diluted suspensions of bacteria shown by at least three independent experiments (Supplementary Figure). Since the concentration of bacteria lies within the linear range of a standard curve, it would be adequate to use absorbance to estimate CFU/mL.

**Figure 1:**
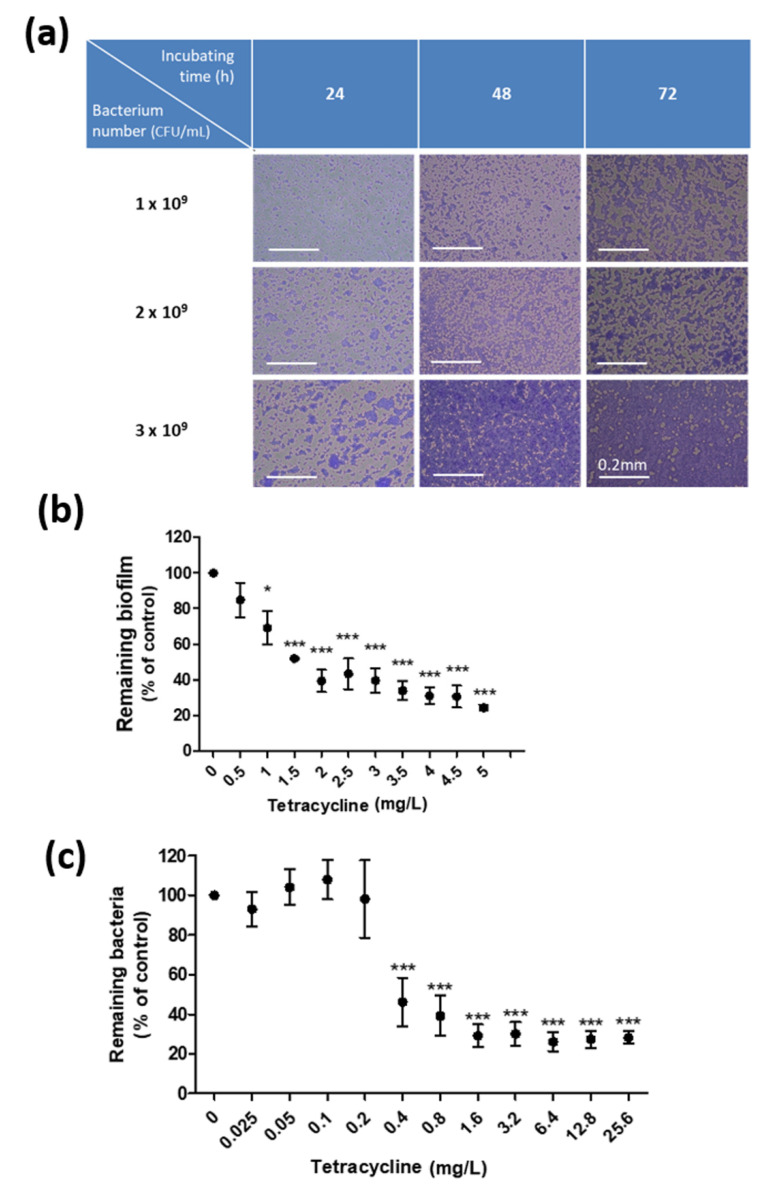
Development of an in vitro culture system for biofilms of *Cutibacterium* (*C.*) *acnes*. (**a**) Biofilms of *C. acnes* were cultured in vitro by incubating the bacterium with 0.5% dextrose glucose for 24–72 h. Original magnification, 200x. Scale bar = 0.2 mm. (**b**) The biofilm of *C. acnes* cultured in vitro was treated with tetracycline (TCN) at indicated dosages. The minimal dose of TCN that reduced the biofilm of *C. acnes* by 50% was 1.5 mg/L; *n* = 3 (mean ± SEM, * *p* < 0.05, *** *p* < 0.001). (**c**) A bacterial suspension of *C. acnes* was treated with TCN at the indicated dosages. The minimal dose of TCN that reduced a suspension of *C. acnes* by 50% was 0.4 mg/L; *n* = 3 (mean ± SEM, * *p* < 0.05, *** *p* < 0.001).

**Figure 2:**
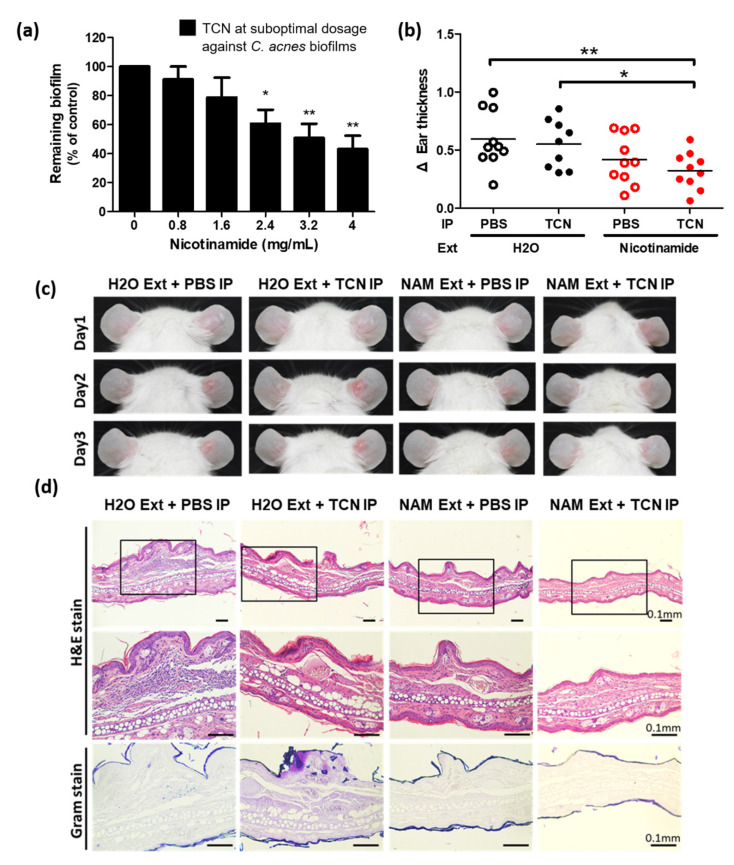
Nicotinamide (NAM) potentiates the efficacy of a suboptimal dose of tetracycline (TCN) against *Cutibacterium* (*C.*) *acnes*. (**a**) The biofilm of *C. acnes* was treated with a combination of TCN and NAM at indicated dosages in vitro. Note that the dosage of TCN (1.2 mg/L) was suboptimal to reduce the biofilm by 50% (see Figure 1b). *n* = 4 (mean ± SEM, * *p* < 0.05, ** *p* < 0.01). (**b**) Mice inoculated with *C. acnes* were treated with combinations of topical NAM and/or intraperitoneal TCN. IP, intraperitoneal injection; Ext, external application; PBS, phosphate-buffered saline; H_2_O, distilled water. The difference in ear thickness on day 3 was analyzed; *n* = 10 in each group (Each dot represents data from a mouse within each group. The horizontal line shows the mean of each group, * *p* < 0.05, ** *p* < 0.01) (**c**) Representative images showing the response of the ears to each treatment for 3 days. (**d**) Histology with hematoxylin and eosin staining and Gram staining demonstrating the swelling, inflammatory infiltration, and abscess formation of the lesional ears on day 3. Original magnification, upper row, 100×; middle and lower rows, 200×. Scale bar = 0.1 mm.

**Figure 3:**
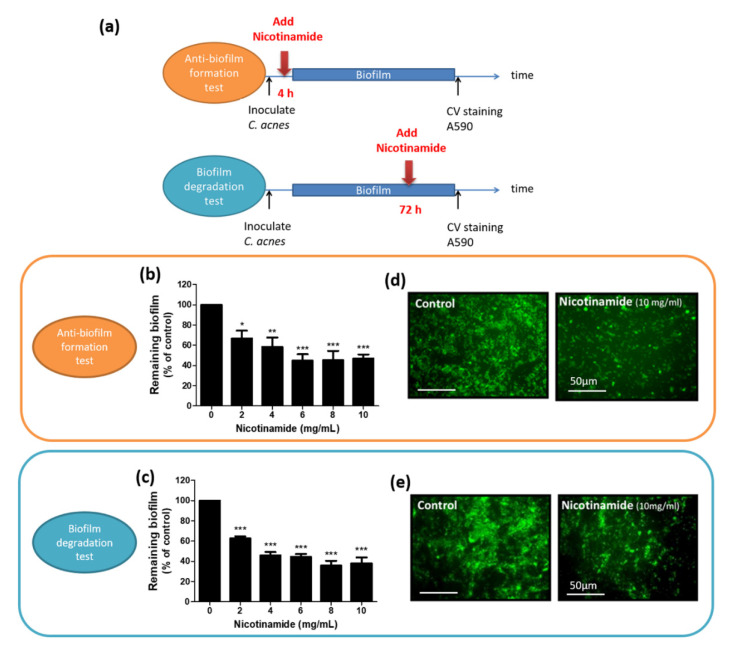
Nicotinamide (NAM) alone inhibits the biofilm of *Cutibacterium* (*C*.) *acnes* in vitro. (**a**) Scheme showing the design of the antibiofilm formation test and biofilm degradation test used in our study. (**b**,**c**) NAM was added to the biofilm at the indicated time post-inoculation of *C. acnes* to better evaluate the effect at specific stages of biofilm development. (**b**) The effect of NAM against biofilms of *C. acnes* shown by the antibiofilm formation test. *n* = 3 (mean ± SEM, * *p* < 0.05, ** *p* < 0.01, *** *p* < 0.001). (**c**) The breakdown of preformed biofilms by NAM in the biofilm degradation test. *n* = 3 (mean ± SEM, * *p* < 0.05, ** *p* < 0.01, *** *p* < 0.001). (**d**,**e**) To visualize the biofilm, the remaining biofilm was stained with FITC and observed under fluorescence microscopy. (**d**) Representative images of fluorescence microscopy of the antibiofilm formation test. Original magnification, 600×. Scale bar = 50 μm. (**e**) Representative images of fluorescence microscopy of the biofilm degradation test. Original magnification, 600×. Scale bar = 50 μm.

*Cutibacterium acnes* in the logarithmic growth phase was serially diluted in BHI supplemented with 0.5% dextrose glucose in the experiments in Figure 1a and diluted to obtain an absorbance of 1.5 at 600 nm (A600 = 1.5, corresponding to 2.7 × 10^9^ CFU/mL of *C. acnes*) in the biofilm tests in Figure 1b, Figure 2a, Figure 3b,c and Figure 5. A bacterial suspension (450 μL) was added to the wells of a 24-well, tissue-culture-treated, flat-bottom plate (Greiner Bio-one, Neuburg, Germany). In indicated experiments, 50 μL of test substances specified below were added to the biofilm at specified time points. In the antibiofilm formation test, *C. acnes* was treated at the early stage of biofilm development (4 h post-inoculation). In the biofilm degradation test, *C. acnes* was treated at the late stage of biofilm development (72 h post-inoculation). For the experiment in Figure 1b, TCN ranging from 0.5 to 5 mg/L was added at 72 h after the start of incubation. For the experiment in Figure 2a, NAM ranging from 0.8 to 4 mg/mL was given in addition to 1.2 mg/L TCN at 4 h after the start of incubation. For the experiments in Figure 3b,c, NAM ranging from 2 to 10 mg/mL was added at 4 h and 72 h post-inoculation, respectively. For the experiment shown in Figure 5, 0.25, 0.5, and 1 unit/mL of DNase I was given in combination with 4 mg/mL NAM at 4 h after the start of incubation.

After a total incubation of 72 to 96 h, spent media and free-floating bacteria were removed by aspiration, and biofilms were left on the bottom of the plates. To quantify the remaining biofilm, a biofilm-coated plate was first washed with phosphate-buffered saline (PBS), fixed with methanol for 5 min, and dried thoroughly. The biofilm was next stained with 200 μL of 0.3% crystal violet for 20 min, washed to remove unbound dye, dried again, dissolved with 400 μL of 30% (*v/v*) acetic acid and transferred to wells of a 96-well plate. The quantity of remaining biofilm was determined by the absorbance at 590 nm (A590). All experiments were performed in triplicate, each being repeated at least three times.

**Figure 4:**
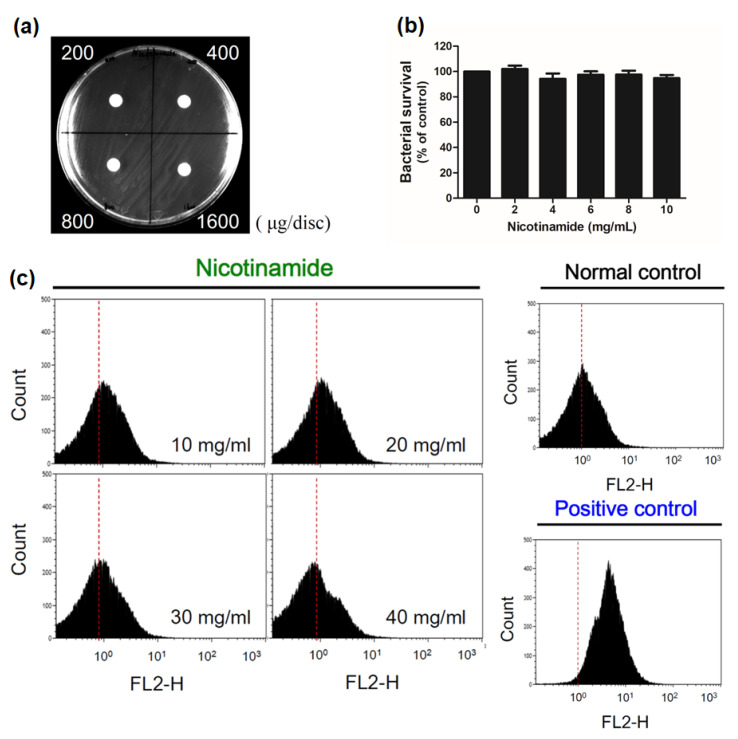
Nicotinamide (NAM) shows no antibacterial activity at the tested dosages. (**a**) An agar diffusion test showing that NAM did not inhibit the growth of *Cutibacterium* (*C.*) *acnes* at dosages of <1600 μg/disc. (**b**) A bacterial suspension of *C. acnes* was treated with NAM at the indicated dosages. No effect on bacterial growth was detected at dosages of <10 mg/mL; *n* = 3 (mean ± SEM). (**c**) Flow cytometry with propidium iodide staining did not show a shift to the right, suggesting that there was no remarkable cell death after NAM treatment at dosages of <40 mg/mL. Normal control: *C. acnes* with no treatment. Positive control: *C. acnes* exposed to high temperature (75 °C) for 30 min.

Agar Diffusion Test and Microdilution Test: In agar diffusion tests, NAM-containing paper discs at dosages of 200, 400, 800, and 1600 μg/disc were placed in the center of BHI agar plates that were inoculated with *C. acnes* and incubated for 72 h. In microdilution tests of Figure 1c or Figure 4b, serially diluted TCN ranging from 0.025 to 25.6 mg/L or NAM ranging from 2 to 10 mg/mL was plated into a sterile 96-well plate at 190 μL. Ten microliters of a *C. acnes* suspension of an absorbance of 0.5 at 600 nm (A600 = 0.5, approximately 7.1 × 10^8^ CFU/mL of *C. acnes*) was then added to each well and incubated for 24 h. Bacterial survival was measured by the absorbance at 600 nm (A600). All experiments were performed in triplicate, each being repeated at least three times.

Animals and the Acne Animal Model: The animal study in this article has been reviewed and approved by the institutional Animal Care and Use Committee at Taipei Medical University (LAC-2017-0081, 19 April 2017). *Cutibacterium acnes* was inoculated into the ears of mice as previously described [24]. Briefly, 10 μL of *C. acnes* at 5 × 10^9^ CFU/mL was injected intradermally into the right ear of 6-week-old ICR mice, and PBS was injected into the left ear as a control. In the indicated group, intraperitoneal injection of TCN was given at a dosage of 1.5 mg/kg body weight once a day, and paper discs were soaked in 40 mg/mL of NAM and applied topically to the ears of the mice for 10 min twice a day. The ear thickness was measured daily with microcalipers for 3 days. The difference in ear thickness was calculated by subtracting the thickness of the control ear from that of the lesional ear of the same mouse. On day 3, the mice were sacrificed, and the lesional ears were excised and sent for pathology, H&E staining, and Gram’s staining.

Fluorescence Microscopy: Log-phase *C. acnes* was suspended to an A600 reading of 1, corresponding to 1.7 × 10^9^ CFU/mL of *C. acnes*, in BHI supplemented with 0.5% dextrose glucose. The bacterial suspension with or without 10 mg/mL NAM was added to wells of a sterile eight-well chamber slide (Thermo, Rochester, NY). After a 72 h incubation, spent medium and free-floating bacteria were removed, and the wells were washed with PBS, fixed, and stained with 200 μL FITC (0.1% (*w/v*) FITC in PBS) for 20 min in a dark environment. The wells were then washed with PBS and dried thoroughly, and the chambers were removed. The slides were preserved with FluorSave Reagent, mounted using standard methods, and examined with a fluorescence microscope.

Flow Cytometric Analysis: A suspension of *C. acnes* was treated with 10, 20, 30, and 40 mg/mL of NAM for 30 min and then incubated with PI for 15 min at 37 °C. The fluorescence intensity of PI was measured using a flow cytometer (FACScan, Becton Dickinson, Franklin Lakes, NJ, USA).

Computational Molecular Docking: The crystal protein structure of DNase I was retrieved from the Protein Data Bank, with a corresponding Protein Data Bank ID of 4AWN [25]. AutoDock 4.2 was used to calculate the docking positions on DNase I for NAM and N-acetylglucosamine and their respective binding energies [26]. Totally, 2.5 × 10^6^ evaluations and 100 runs of docking were performed following the Lamarckian genetic algorithm. The docking position and the lowest binding energy were visualized by the PyMol Molecular Graphics System (Schrödinger, New York, NY, USA).

Surface Plasmon Resonance: The binding kinetics between NAM and DNase I were assessed using a BIAcore T200 model (GE Healthcare Bio-Sciences Corp., Piscataway, NJ, USA) with a CM5 sensor chip at 25 °C in HBS-EP buffer (0.01 M HEPES (pH 7.4), 0.15 M NaCl, 3 mM EDTA, and 0.005% *v/v* surfactant P20; GE Healthcare). The flow system was primed three times before initiation. Indicated concentrations of NAM were injected into the DNase I–immobilized sensor chip at a flow rate of 30 μL/min for 120 s and allowed to dissociate for 60 s. T-200 BIA evaluation software (GE Healthcare) was used to subtract references and determine the steady-state equilibrium dissociation constant.

Enzymatic Assay of DNase I: This assay was modified from procedures described in previous studies [27,28]. Briefly, 150 μL of DNA substrate (66 μg/mL in reaction buffer) with or without NAM was added to a 96-well flat-bottom ultraviolet-transparent plate (Costar, Corning, NY, USA) on ice. Then 30 μL of DNase I (400 Kunitz units/mL in reaction buffer) was mixed with DNA substrate immediately before the recording began. The absorbance at 260 nm (A260) was measured at 5 min intervals for 30 min. All experiments were repeated at least three times.

**Figure 5:**
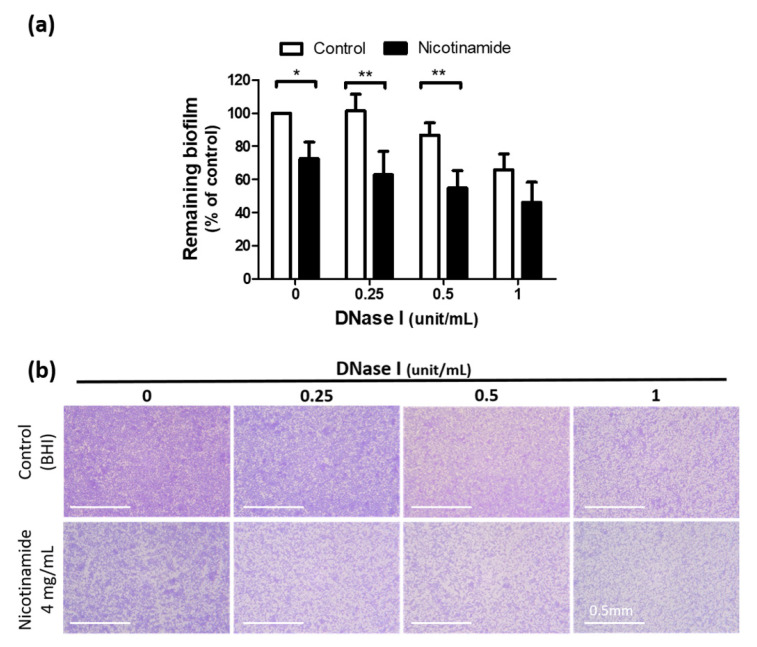
Nicotinamide (NAM) enhances the antibiofilm effect of deoxyribonuclease (DNase) I against *Cutibacterium* (*C*.) *acnes*. (**a**) The biofilm of *C. acnes* was treated with DNase I at the indicated concentrations in combination with NAM or the control. *n* = 5 (mean ± SEM, * *p* < 0.05, ** *p* < 0.01). (**b**) Representative images showing the effect of DNase I against the biofilm of *C. acnes* with or without NAM. Original magnification, 100×. Scale bar = 0.5 mm. BHI, brain heart infusion.

Statistical Analysis: The *p* values in Figure 1b,c, Figure 2a, Figure 3b,c and Figure 4b were calculated from a repeated-measures analysis of variance (ANOVA) test with Dunnett’s correction. The *p* values in Figure 2b or Figure 5a were calculated from a two-way ANOVA test with the Bonferroni correction. All significance levels were set to *p* < 0.05.

## 3. Results

### 3.1. Establishment of an In Vitro Culture System for Biofilms of C. acnes

To evaluate the effect of NAM on biofilms of *C. acnes*, we first modified a previously reported protocol to obtain biofilms of *C. acnes* in vitro [23]. Figure 1a shows biofilms of *C. acnes* harvested from different concentrations of the bacterium that were cultured for 24, 48, and 72 h in BHI with supplemental dextrose glucose. In all the following biofilm tests, we chose to incubate 2.7 × 10^9^ CFU/mL of *C. acnes* (A600 = 1.5) for 72 h to yield biofilms.

One of the universal features of biofilms is their relative resistance to antibiotics [13]. Consistent with that, Figure 1b,c shows that biofilms harvested from this culture system require more than 3.75-fold TCN to be inhibited by 50%, compared to the dosage required for their suspended counterpart. The 50% minimum biofilm inhibitory concentration (MBIC_50_) was 1.5 mg/L TCN for biofilms of *C. acnes* (Figure 1b), while the 50% minimum inhibitory concentration (MIC_50_) was 0.4 mg/L TCN for the suspensions (Figure 1c). In other words, although TCN at a dosage over 0.4 mg/L is sufficient to reduce suspensions of *C. acnes* by 50%, it is suboptimal to reduce biofilms of *C. acnes* to the same extent, which required a dose of TCN of >1.5 mg/L.

### 3.2. NAM Potentiates the Efficacy of the Suboptimal Dosing of TCN against C. acnes

To recapitulate the features of TCN-resistant *C. acnes*, we cultured *C. acnes* into biofilms and treated them with a suboptimal dosage of TCN (1.2 mg/L) that was larger than MIC_50_ (0.4 mg/L) but less than MBIC_50_ (1.5 mg/L). Importantly, TCN at 1.2 mg/L was enough to inhibit suspensions of the bacterium but suboptimal to inhibit its biofilms by itself. In this condition, NAM significantly increased the efficacy of TCN against the biofilm of *C. acnes* (43% ± 9% remaining biofilm under 4 mg/mL NAM in addition to TCN, *p* < 0.01) (Figure 2a), supporting its clinical effectiveness as an adjunctive treatment for acne vulgaris [19].

Next, we investigated the effect of NAM on the efficacy of TCN against *C. acnes* in an animal model of acne vulgaris [24]. *Cutibacterium acnes*–inoculated mice were divided into four groups (*n* = 10 in each group) according to the treatments they received, which were topical treatments with NAM or distilled water crossed with intraperitoneal injections of low-dose TCN or PBS. While an injection of low-dose TCN alone did not relieve ear swelling (0.626 ± 0.076 mm in intraperitoneal TCN plus topical distilled water group versus 0.769 ± 0.065 mm in intraperitoneal PBS plus topical distilled water control group, *p* ≥ 0.05), low-dose TCN markedly reduced the swelling and inflammation when used in combination with topical NAM (0.448 ± 0.029 mm in the intraperitoneal TCN plus topical NAM group versus 0.769 ± 0.065 mm in the intraperitoneal PBS plus topical distilled water control group, *p* < 0.01) (Figure 2b). Figure 2c,d demonstrates the clinical and pathological responses to each treatment in vivo.

### 3.3. Nicotinamide Alone Suppresses Biofilms of C. acnes In Vitro

On top of these results, we further assessed whether NAM inhibited the biofilm of *C. acnes* as a single agent and whether NAM only acted at a specific stage of biofilm development.

As we have mentioned earlier, biofilms of *C. acnes* were well-developed at 72 h post-inoculation of the bacterium using this biofilm culture system. To better evaluate the effect of NAM at the early or late stages of biofilm development, we treated the biofilm at 4 or 72 h post-inoculation of *C. acnes*, as depicted in Figure 3a. Results showed that NAM not only inhibited the formation of biofilm in *C. acnes* at the early stage (58% ± 9% remaining biofilm under 4 mg/mL NAM compared to control, *p* < 0.01) (Figure 3b), but it also enhanced degradation of the preformed biofilm in the late stage (46% ± 3% remaining biofilm under 4 mg/mL NAM compared to control, *p* < 0.001) (Figure 3c). Fluorescent microscopic studies also confirmed these findings (Figure 3d,e).

Some might argue that NAM reduces the biofilm of *C. acnes* by being bacteriostatic or bactericidal itself. To clarify this, a disc diffusion test (Figure 4a) and a microdilution test (Figure 4b) were performed to evaluate the antibacterial activity of NAM against *C. acnes*. Neither of the tests revealed any detectable inhibition of bacterial growth when the concentration of NAM was <10 mg/mL. Furthermore, a flow cytometric analysis of *C. acnes* demonstrated that treatment with NAM, even up to 40 mg/mL, did not induce significant apoptotic cell death (Figure 4c). These results all suggest that NAM had no antibacterial activity at the tested dosages.

### 3.4. Nicotinamide Activates DNase I and Enhances the Antibiofilm Effect of the Enzyme against C. acnes

Since DNase I markedly reduces biofilms in various pathogens [13,17], we next examined whether DNase I suppressed the biofilm of *C. acnes*. Figure 5 shows that DNase I tended to reduce the biofilm of *C. acnes* in our culture system, although this trend did not reach statistical significance. Notably, the antibiofilm effect of DNase I was more prominent in the presence of NAM (87% ± 9% remaining biofilm under 0.5 unit/mL DNase I versus 55% ± 13% remaining biofilm under the same dosage of DNase I plus 4 mg/mL NAM, *p* < 0.01) (Figure 5).

Based on this observation, we assumed that the effect of DNase I on biofilms of *C. acnes* may be regulated by NAM. This assumption was supported by the following facts. First, NAM is a component of two key coenzymes, namely nicotinamide adenine dinucleotide and nicotinamide adenine dinucleotide phosphate, which were shown to bind and interact with approximately 500 human enzymes [29]. Second, the binding between NAM and protein enzymes is not conservative [30], with numerous currently unknown protein–ligand interactions still waiting to be explored.

**Figure 6:**
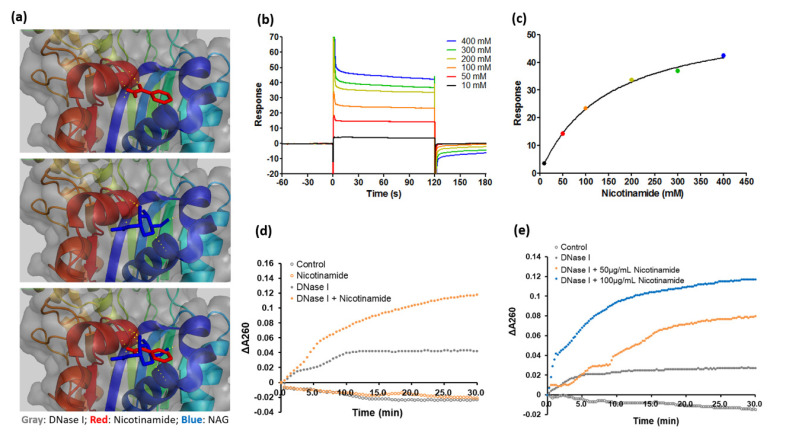
Binding of nicotinamide (NAM) to deoxyribonuclease (DNase) I increases the activity of the enzyme. (**a**) The binding site of NAM (red) and the glycosylation site of N-acetylglucosamine (NAG, blue) on DNase I (gray) are shown by model-assisted computational molecular docking. (**b**) The association and dissociation between NAM and DNase I are shown by surface plasmon resonance. The process was expressed as the differential response unit between the binding of NAM to the DNase I–immobilized sensor chip or to a blank sensor chip. (**c**) The equilibrium-state response unit recorded by surface plasmon resonance is plotted versus the concentration of NAM. The apparent equilibrium dissociation constant is 0.1424 M. (**d**,**e**) The activity of DNase I was measured by a kinetic ELISA, with DNA as substrates. The breakdown of DNA was detected by the absorbance at 260 nm (A260). The slope of the initial curve corresponds to the reaction rate of DNase I. (**d**) Kinetic ELISA showing the effect of NAM on the activity of DNase I. (**e**) Kinetic ELISA depicting the activity of DNase I at indicated dosages of NAM.

As NAM binds to diverse binding motifs across different protein families, next we analyzed the protein–ligand interaction between NAM and DNase I. Figure 6a shows the binding site of NAM (red) on DNase I (gray) identified by computational molecular docking. The site of DNase I where NAM binds—arginine 18—is also a potential site for N-acetylglucosamine glycosylation (blue) [25] (Figure 6a). The affinity and kinetics of the binding were determined by a surface plasmon resonance analysis. The association and dissociation curves demonstrated an apparent equilibrium dissociation constant of the binding of 0.1424 M (Figure 6b,c). As the binding of NAM to DNase I does not necessarily justify an increase of the enzymatic activity, the activity of DNase I with or without the presence of NAM was determined by an enzymatic kinetic assay. Briefly, the alteration of absorbance at 260 nm that corresponded to the rate of digestion of DNA substrate was measured by a spectrophotometer over a defined time interval [27,28]. The enzymatic kinetic assay showed that NAM accelerated the reaction of DNase I dose dependently (Figure 6d,e), further supporting that NAM activated DNase I through the binding to the enzyme.

## 4. Discussion

**Figure 7:**
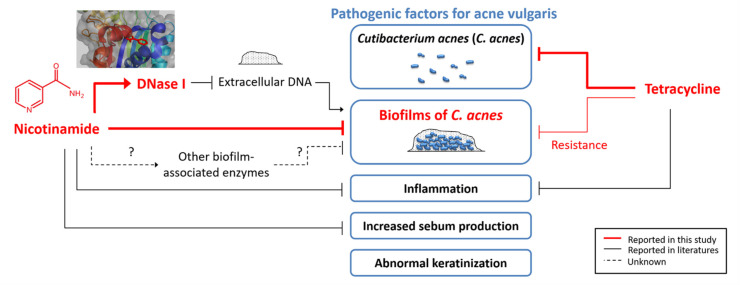
Diagram summarizing the mechanism of action of nicotinamide (NAM) in acne vulgaris.

Expanding the knowledge on the effect of NAM on biofilms of *C. acnes* is a first step toward new directions for investigation to better combat acne vulgaris. NAM is known to be anti-inflammatory and sebo-suppressive in the literature [31]. This study adds information that NAM, as a single agent, is able to inhibit the biofilm of *C. acnes* to some extent. We have shown that NAM not only enhanced the performance of suboptimal dosing of TCN against *C. acnes* in mice but also inhibited the biofilm of *C. acnes* in a biofilm culture system. Moreover, we have proposed a mechanism for the antibiofilm effect by showing an interaction between NAM and DNase I. Although future work needs to be done to determine whether NAM regulates other enzymes that are associated with the formation or degradation of biofilms, this study has validated the role of NAM as an adjunctive treatment for acne vulgaris. Figure 7 summarizes the mechanism of action of NAM in acne vulgaris that was supported by this and other studies [7,17,19,22,31,32].

In the acne animal model, we have demonstrated that NAM potentiated the effect of TCN. However, we did not find the direct association between NAM and TCN. Instead, our findings have suggested that NAM might improve the effect of TCN through the inhibition of formation of biofilms in *C. acnes*. Biofilm formation has been proved to decrease the penetration of TCN and various antibiotics and to associate with drug resistance in chronic infections [13]. As our results showed that NAM hampered the formation of biofilms of *C. acnes*, more *C. acnes* would be kept in a planktonic lifestyle. This provides a reason why TCN at the same low dosage could better inhibit the bacteria in the presence of NAM in vivo. Importantly, even in the presence of very high concentration of NAM (4 mg/mL) in vitro, about 50% of biofilms still exist. One of the explanations is that NAM has minimal cytotoxicity [19] and, thus, is not bacteriostatic or bactericidal in nature. On top of this, it should be more reasonable to use NAM in combination with antibiotics or other anti-acne drugs in clinical settings. Echoing our findings, a recent study has demonstrated that NAM effectively suppressed biofilm formation and exhibited significant antifungal activity against fluconazole-resistant *Candida albicans* [33].

Our results have suggested that NAM inhibits biofilms of *C. acnes* by indirectly targeting extracellular DNA, which is a promising strategy to fight against biofilm-related chronic cutaneous infections. Extracellular DNA is essential for biofilms. A recently published study by Doroshenko et al. indicated that extracellular DNA interferes with the penetration of antibiotics into this fortress [34]. Examples of therapeutic agents that target extracellular DNA are DNase I [18], DNase-coated nanoparticles [35], and monoclonal antibodies [36].

Although DNase I is known to target extracellular DNA, interestingly, we have showed that exogenous DNase I is not absolutely necessary for NAM to inhibit *C. acnes* biofilms. The reason is that an endogenous source of DNase I exists. Several early studies indicate that DNases are present in human acne lesions but work inefficiently. The production of DNases has been reported to increase in *Cutibacterium* strains from acne patients [37]. However, only 30% of the DNases from these *C. acnes* strains possess positive or weak enzymatic reactions [38]. According to the above literature, one of the explanations of our result is that NAM activates the endogenous DNases, which are produced by *C. acnes* strains but work inefficiently at baseline, to achieve the antibiofilm effects.

Many studies have provided evidence supporting the claim that NAM is able to activate DNase I. First, NAM was reported to increase DNase I activity in an inflammatory skin mouse model [39]. Second, the site where NAM binds on DNase I is also a potential site for N-acetylglucosamine glycosylation of the enzyme [25]. Glycosylation of this specific site is associated with full activity, heat-stability, and trypsin resistance [40]. Thus, through occupying arginine 18 of DNase I, NAM may upregulate the activity of DNase I as N-acetylglucosamine glycosylation does.

Besides targeting extracellular DNA, several other antibiofilm strategies were proposed against *C. acnes* and other pathogens. These include interfering matrix compound synthesis, enhancing matrix degradation, and applying surfactants to surfaces [41]. We have reviewed published antibiofilm treatments against *C. acnes*. However, some of them are still in the early stage of research and lack clinical supporting data, such as thiazolidinedione derivatives (quorum-sensing inhibitors) [42], and some of them are surfactants that possibly cause skin irritation after frequent contact [43], such as decanediol [44]. Still others claim an antibiofilm effect without investigating the underlying mechanism or showing the risk of allergy and irritancy, for example, using plant extracts (*Epimedium brevicornum* [45], *Polygonum cuspidatum* [45], *Myrtus communis* [46], and *Rhodomyrtus tomentosa* [47]) and chicken egg yolk antibodies [48].

As drug repurposing stays on-trend, more and more new pharmacological applications are identified for already approved drugs [49]. One of the examples is the combination of antibiotics and non-antibiotic compounds that could inhibit bacterial resistance determinants or enhance antibiotic activity in confronting multidrug-resistant bacteria [50]. Many antibiotics could overcome bacterial resistance when used in combination with adjuvants, such as resistance inhibitors, membrane saboteurs, signaling inhibitors, or immune enhancers [50]. We believe that NAM, with a well-established safety profile [19] and an antibiofilm effect proposed by this study, could be an ideal adjuvant treatment for attacking resistant *C. acnes*.

One limitation of our study is that the effects of NAM on enzymes other than DNase I were not thoroughly investigated. It is also possible that NAM reduces biofilms of *C. acnes* through other mechanisms, such as increasing their dispersal. As the formation and degradation of biofilms are associated with a suite of enzymes [41], whether NAM regulates any of these should be assessed in future studies. Additionally, factors other than the biofilm may also contribute to the persistence of acne vulgaris, such as increased sebum production [51,52]. By inhibiting sebum production, an acetyl coenzyme A carboxylase inhibitor showed very promising results against acne vulgaris [53]. As NAM is a known sebo-suppressive agent [31], the effects of NAM on enzymes involved in sebum production and lipid metabolism are also worth exploring.

## 5. Conclusions

Nicotinamide enhances the activity of DNase I to attenuate biofilms of *C. acnes*. Despite its exploratory nature, this study proposes a potential novel strategy to fight against *C. acnes* and other biofilm-related chronic cutaneous infections in the future.

## Data Availability

All data generated or analyzed during this study have been included in this article.

## Supplementary Materials

The following are available online at https://www.mdpi.com/article/10.3390/pharmaceutics13060819/s1, Figure S1: the Correlation between the Concentration of Bacteria Suspension of *Cutibacterium* (*C*.) *acnes* and Absorbance at 600 nm by Spectrophotometer.
