# Supplementary material for: Activation of Deoxyribonuclease I by Nicotinamide as a New Strategy to Attenuate Tetracycline-Resistant Biofilms of Cutibacterium acnes
Source: Pharmaceutics. 2021 May 31;13(6):819. doi: 10.3390/pharmaceutics13060819 (PMC8228415; doi:10.3390/pharmaceutics13060819)
Supplement: Supplementary file 1 [file pharmaceutics-13-00819-s001.zip › pharmaceutics-1239059-supplementary.pdf]

pharmaceutics

MDPI

Supplementary Materials: Activation of Deoxyribonuclease I by Nicotinamide as a New Strategy to Attenuate Tetracycline-Resistant Biofilms of *Cutibacterium acnes*

Yi-Hsien Shih, Donald Liu, Yen-Chou Chen, Ming-Hsuan Liao, Woan-Ruoh Lee and Shing-Chuan Shen

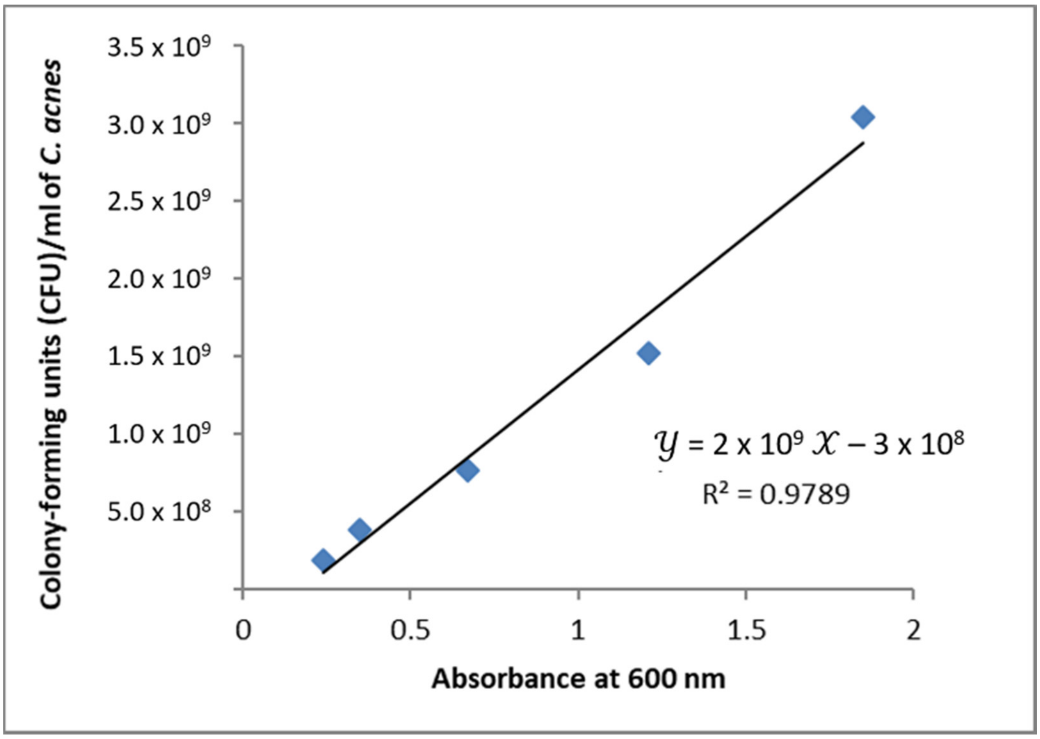

Figure S1. The Correlation between the Concentration of Bacteria Suspension of *Cutibacterium* (*C.*) *acnes* and Absorbance at 600 nm by Spectrophotometer.
